# TRPM4 in Cancer—A New Potential Drug Target

**DOI:** 10.3390/biom11020229

**Published:** 2021-02-05

**Authors:** Anna Borgström, Christine Peinelt, Paulina Stokłosa

**Affiliations:** National Center of Competence in Research NCCR TransCure, Institute of Biochemistry and Molecular Medicine, University of Bern, 3012 Bern, Switzerland; christine.peinelt@ibmm.unibe.ch (C.P.); paulina.stoklosa@ibmm.unibe.ch (P.S.)

**Keywords:** ion channel, cancer, drug target, proliferation, migration, calcium, prognostic marker

## Abstract

Transient receptor potential melastatin 4 (TRPM4) is widely expressed in various organs and associated with cardiovascular and immune diseases. Lately, the interest in studies on TRPM4 in cancer has increased. Thus far, TRPM4 has been investigated in diffuse large B-cell lymphoma, prostate, colorectal, liver, breast, urinary bladder, cervical, and endometrial cancer. In several types of cancer TRPM4 is overexpressed and contributes to cancer hallmark functions such as increased proliferation and migration and cell cycle shift. Hence, TRPM4 is a potential prognostic cancer marker and a promising anticancer drug target candidate. Currently, the underlying mechanism by which TRPM4 contributes to cancer hallmark functions is under investigation. TRPM4 is a Ca^2+^-activated monovalent cation channel, and its ion conductivity can decrease intracellular Ca^2+^ signaling. Furthermore, TRPM4 can interact with different partner proteins. However, the lack of potent and specific TRPM4 inhibitors has delayed the investigations of TRPM4. In this review, we summarize the potential mechanisms of action and discuss new small molecule TRPM4 inhibitors, as well as the TRPM4 antibody, M4P. Additionally, we provide an overview of TRPM4 in human cancer and discuss TRPM4 as a diagnostic marker and anticancer drug target.

## 1. Introduction

Transient receptor potential (TRP) family is a large superfamily of widely expressed ion channels, which mainly conduct cations, including Ca^2+^, Mg^2+^, and Na^+^. TRP channels are responsible for a broad range of cellular functions, and many of these channels regulate intracellular Ca^2+^ homeostasis and signaling. TRP channels are divided into six mammalian subfamilies based on their structural homology, and each family differs in its mode of activation [1].

Transient receptor potential melastatin 4 (TRPM4) belongs to the TRPM channel subfamily. TRPM4 and TRPM5, the closest homologue of TRPM4, differ from the other TRP family members because they do not conduct Ca^2+^ but only monovalent cations [2,3]. Intracellular Ca^2+^ directly activates TRPM4, which then conducts an influx of Na^+^ [4]. In 2017 and 2018, almost simultaneously, four independent groups published cryo-EM structures of TRPM4, revealing both a nucleotide-binding domain (NBD) that binds ATP and is located in the N-terminal and a Ca^2+^ binding site in the transmembrane domain [5,6,7,8]. The Ca^2+^-activated activity of TRPM4 can be modified by ATP, IP_3_, calmodulin, and protein kinase C-dependent phosphorylation [9,10]. TRPM4 was shown to be voltage-dependent, as membrane potential strongly modulates its activity [11]. Positive potential increases TRPM4 conductivity. However, changes in membrane potential are not sufficient to activate the channel.

TRPM4 is widely expressed in various organs, although its expression is the highest in the prostate, colon, and heart [2,11]. TRPM4 is also expressed in immune cells (dendritic, mast, and Th1 and Th2 cells) [12,13,14], as well as in the central nervous system [15]. Hence, TRPM4′s broad expression patterns support its potential implications in the physiological functions of different cells, tissues, and organs. Indeed, TRPM4 has been linked to a range of physiological processes, many involving fundamental cellular functions. Dysregulations of TRPM4 by either altered expression levels or mutations have been linked to several pathological conditions, including cardiac disorders [16,17,18,19,20,21,22,23,24], immune diseases [13,14,25,26], and neurological disorders [27,28,29]. In addition, TRPM4 was suggested to be an interesting pharmacological target for the treatment of mucus-related diseases, such as cystic fibrosis, as it was recently shown to be involved in goblet cell mucin secretion [30]. TRPM4 has been suggested to play a role in insulin secretion in pancreatic β-cells [31]. However, another study using TRPM4 knockout mice reported no differences in glucose-induced insulin release compared to wild-type mice [14]. Lately, growing interest has been directed toward TRPM4 and its role in several types of cancer [32,33,34,35,36,37,38,39,40,41]. TRPM4 contributes to several cancer hallmark functions, such as cell migration, proliferation, and the epithelial to mesenchymal transition (EMT). In this paper, we review the emerging data about TRPM4′s involvement in cancer hallmark functions, as well as the correlation of TRPM4 expression with patient outcome. We summarize the possible mechanisms of action, such as altered intracellular Ca^2+^ signaling, covalent modifications (glycosylation and phosphorylation), and protein interaction partners and discuss recent advances with new TRPM4 blockers, including the M4P antibody and small molecule inhibitors.

## 2. General Mechanisms of TRPM4

Upon activation, TRPM4 conducts Na^+^ ions into the cell. This influx of positive ions depolarizes the plasma membrane and thereby decreases the driving force for Ca^2+^ entry via store-operated Ca^2+^ entry (SOCE) and other Ca^2+^ entry pathways [42]. SOCE has been linked to several fundamental cellular processes, including gene expression. Alterations in SOCE Ca^2+^ signaling were shown to contribute to several cancer hallmark functions, e.g., decreased apoptosis and increased proliferation and migration [43,44,45,46,47,48,49,50,51]. In nonexcitable cells, SOCE is the main Ca^2+^ entry pathway. A plethora of extracellular stimuli induce intracellular IP_3_ production and, subsequently, endoplasmic reticulum (ER) Ca^2+^ store depletion. The drop in ER luminal Ca^2+^ concentration sequentially activates the unfolding of stromal interaction molecule 1 (STIM1) and, subsequently, activates the store-operated Orai1 Ca^2+^ channels [52]. Anticancer drugs may involve changes in Ca^2+^ signaling [53,54]; moreover, membrane transport proteins contribute to chemoresistance in several types of cancer [55]. Although TRPM4 is nonpermeable to Ca^2+^, Na^+^ influx via TRPM4 decreases membrane potential and results in a decrease in intracellular Ca^2+^ signaling in many different cells, including rat dental pulp stem cells, various immune cells, and cancer cells [2,12,13,14,41,56,57]. However, an increase in SOCE by TRPM4 expression has also been observed [35,58]. Clearly, a mechanism fundamentally altering intracellular Ca^2+^ signaling broadly affects cellular functions and, if dysregulated, adds to cellular malfunctions. In line with this, TRPM4 has been described to be involved in the Ca^2+^-dependent migration of vascular endothelial cells [59], dendritic cells [13], mast cells [26], and T-cells [12], as well as T-cells’ cytokine production [12,25]. In addition, TRPM4 was shown to colocalize with focal adhesion proteins in mouse embryonic fibroblasts and to regulate focal adhesion turnover, a process that is important for cell migration [60,61].

Cancer-derived alterations in crosstalk between Ca^2+^ and reactive oxygen species (ROS) contribute to changes in Ca^2+^-dependent cancer hallmark functions, such as a decreased ability to induce apoptosis, increased proliferation, migration, and changes in mitochondrial metabolism [62]. ROS impair intracellular Ca^2+^ signaling, as Ca^2+^-transporting membrane enzymes, lipids, and endoplasmic reticulum–mitochondria interfaces depend on ROS [63]. ROS can decrease SOCE via a blockage of STIM1′s homologue ER Ca^2+^ sensor, STIM2 [64]. In contrast to Orai1, its homologue Orai3 is not sensitive to ROS, and the Orai1/Orai3 ratio determines if intracellular SOCE Ca^2+^ signaling depend on ROS [65,66,67,68]. Both STIM2 and Orai3 subunits are dysregulated in different types of cancer and contribute to metastatic spread, proliferation, and reduced apoptosis [43,67,68,69,70,71,72,73,74,75,76,77,78,79,80]. TRPM4 directly contributes to H_2_O_2_-dependent migration, possibly because H_2_O_2_ prevents the desensitization of TRPM4 [59,81]. However, ROS-dependent alterations of TRPM4 activity in cancer cells have not yet been investigated and determining the contributions of TRPM4 to ROS-dependent alterations of Ca^2+^ signaling in cancer will be a challenge in the future.

The glycosylation patterns of membrane proteins are altered in aging processes, cancer, and immune diseases [82]. Changes in complex N-glycosylation can alter the functions of membrane transport proteins, especially ion channels [83,84,85,86,87,88]. Phosphorylation can switch ion channel functions on or off, and the concept of a regulatory mechanism that couples changed phosphorylation patterns in cancer cells to the response or nonresponse of ion channels has long been studied for various ion channels, including TRP channels [89]. TRPM4 is glycosylated at aspartate N992, and glycosylation can stabilize surface expression and reduce TRPM4 currents [90,91]. In addition, other gain-of-function mutations impair TRPM4 surface expression [92,93]. For TRPM4, two phosphorylation sites (Ser1145 and Ser1152) have been identified, and phosphorylation can increase the Ca^2+^ sensitivity of TRPM4 [9]. It has been demonstrated that casein kinase 1 phosphorylation of Ser839 controls the basolateral expression of TRPM4 in smooth muscle cells [94]. Future studies will reveal if the altered glycosylation or phosphorylation patterns of TRPM4 in cancer have pathophysiological implications.

Several interaction partners of TRPM4 have been reported, such as sulfonylurea receptor (SUR1) [29,95,96], 14-3-3 [97], potassium channel tetramerization domain-containing protein 5 (KCTD5) [39], small ubiquitin-related modifier (SUMO) [16,17], TRPC3 [98], protein tyrosine phosphatase non-receptor type 6 (PTPN6) [99], and subunits Glu2A and Glu2B of the N-methyl-D-aspartate receptor (NMDA) receptor [100]. KCTD5 has been shown to correlate with particular classes of breast cancer [39]. In addition, PTPN6 is an unfavorable marker of colorectal cancer (CRC) and has been shown to be involved in the cell cycle regulation and suppression of oncogene β-catenin [101]. Both 14-3-3 and PTPN6 can possibly modify TRPM4 via phosphorylation.

**Figure 1:**
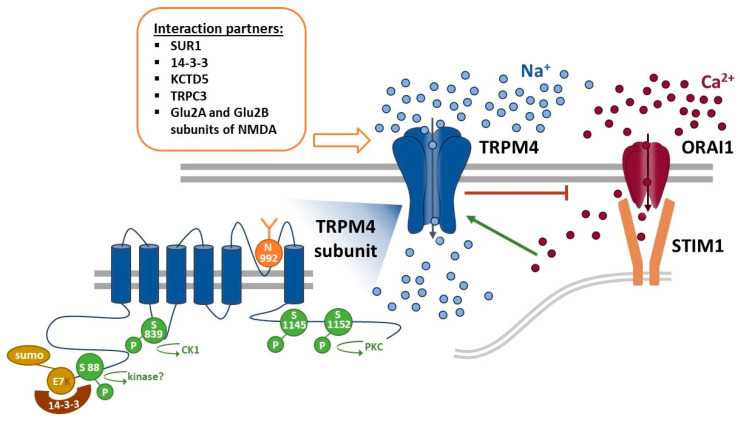
Activation of TRPM4 and the regulation of Ca^2+^ entry. An increase in intracellular Ca^2+^ concentration activates TRPM4, which opens and conducts a large influx of Na^+^ ions. The positively charged Na^+^ ions then depolarize the plasma membrane and thereby inhibit store-operated Ca^2+^ entry (SOCE) via the Orai1 channel. The dysregulation of intracellular Ca^2+^ signaling is linked to several cancer hallmark functions, including increased proliferation, migration, invasion, and the inability to induce apoptosis. TRPM4 has several interaction partners that, in part, have been shown to modulate its functions, including SUR1 [102], 14-3-3 [102], KCTD5 [39], SUMO [16], TRPC3 [102], and subunits Glu2A and Glu2B, which are subunits of the NMDA receptor [100]. In addition, TRPM4 can be glycosylated and phosphorylated.

Figure 1 summarizes the reported mechanisms of action and associated molecular architecture of TRPM4 described in this section, including Ca^2+^ signaling, protein interaction partners, ROS, glycosylation, and phosphorylation. While some of these are under intense investigation (see also Section 4 on the functional role of TRPM4 in different types of cancer), future studies are needed to decipher the underlying mechanisms of the pathophysiology of TRPM4 in cancer.

## 3. TRPM4 Expression in Cancer

**Figure 2:**
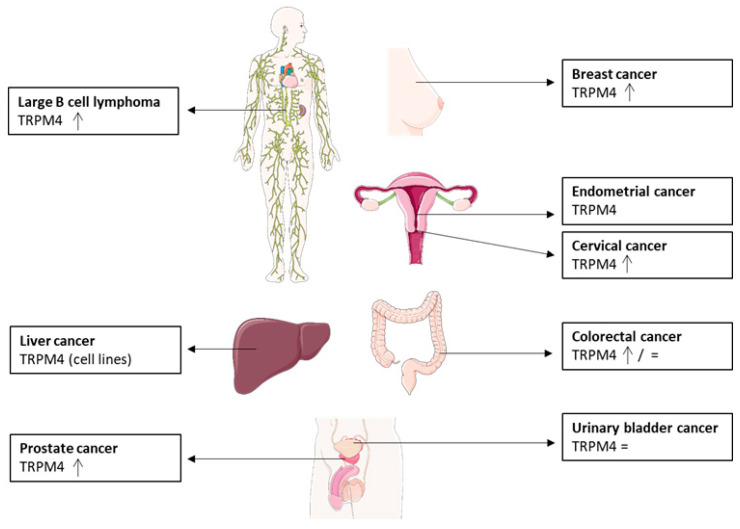
Summary of TRM4 expression levels in different cancer types compared to healthy control tissue (=: expression levels not changed, arrow: increased expression levels) [109].

TRPM4 expression levels have been investigated in a number of different cancers (Figure 2) [32,33,34,38,39,40,41,101,102,103,104,105,106]. In most studies, TRPM4 protein expression levels were reported to be increased in tumor samples compared to healthy tissue. However, in urinary bladder cancer, no changes at the protein or mRNA level have been reported [105]. For colorectal cancer, TRPM4 mRNA expression has been reported to be either decreased or unchanged in comparison to the control tissue [104,107]. Interestingly, most studies on TRPM4 expression in cancer showed changes in the expression levels of TRPM4, but, so far, no TRPM4 mutations have been reported to be associated with cancer. In contrast to cancer, mutations of TRPM4 have also been reported in other pathologies, such as cardiac conduction diseases [16,17,21,108].

In prostate cancer (PCa), TRPM4 protein levels were reported to be increased in cancerous prostate tissue compared to benign glands [103]. In addition, samples with high-intensity TRPM4 staining and an H-score (histological score) equal to or above the median were shown to correlate with an increased risk of biochemical recurrence [103]. In a smaller study of 20 patients, Holzmann et al. reported increased TRPM4 expression in prostatic intraepithelial neoplasia (PIN), which involves tissue transitioning into cancerous tissue, compared to normal prostate tissue [41]. Furthermore, a medium to strong staining intensity of TRPM4 was detected in tissue with an increased Gleason score. However, no significant correlation between clinical parameters or tumor stages was detected. In another study, a significant increase in TRPM4 mRNA expression in tumor samples compared to the controls and increased TRPM4 levels in the less differentiated and more aggressive tissue samples categorized with a higher Gleason score (>7) were reported [34]. Moreover, in a recent study of a tissue microarray from 210 prostate cancer patients, a correlation between TRPM4 protein expression intensities (scores of 3 vs. 4, the second highest and highest TRPM4 scores, respectively) and both local and metastatic progression was described [110].

In two recent studies, TRPM4 was reported to be upregulated in breast cancer samples at both the mRNA and protein levels [39,40]. TRPM4 mRNA was significantly upregulated in breast cancer tissue compared to normal breast tissue [39]. Moreover, a correlation was described between increased histology scores, including poorly differentiated and highly proliferative tissues from intermediate and high-graded breast cancer, and increased TRPM4 expression. Furthermore, in this study, TRPM4 was reported to interact with KCTD5 protein. Both TRPM4 and KCTD5 mRNA expression was shown to be increased in triple negative samples, a more aggressive subtype of breast cancer that lacks specialized treatment [111,112], as well as in ER+/PR+ samples. Additionally, in an independent immunohistochemistry study analyzing 99 breast cancer samples, TRPM4 protein was found to be significantly overexpressed in breast cancer tissue compared to normal breast ducts. The breast cancer tissue displayed TRPM4-specific staining in the cytoplasm and membrane of breast cells, whereas the surrounding stromal cells were negative for TRPM4. Furthermore, increased TRPM4 staining intensity was significantly correlated with a worse prognosis, as demonstrated by a higher lymph node status and a higher pathologic prognostic stage [40].

TRPM4 protein expression was also investigated in diffuse large B cell lymphoma (DLBCL). TRPM4 was mainly detected in the membranes of DLBCL cells, while nonmalignant B cells did not express TRPM4. TRPM4 protein expression was significantly correlated with worse overall survival and worse progression-free survival. In addition, both the analysis of publicly available microarray data and immunohistochemical analysis showed higher TRPM4 expression in the activated B cell-like DLBCL (ABC-DLBCL) subtype, and this increased expression was significantly correlated with worse overall survival in R-CHOP chemotherapy-treated DLBCL samples [33].

Three studies investigated TRPM4 expression levels in colorectal cancer. In 2015, Sozucan et al. reported decreased mRNA expression when 93 CRC patient tissue samples were analyzed [104]. Later, in a transcriptomic-based study comparing the mRNA expression levels of the Ca^2+^ remodeling component between a normal colon cell line and a CRC cell line, no changes in TRPM4 mRNA expression levels were detected [107]. More recently, in an immunohistological study involving a 379 CRC patient tissue microarray, a distinct pattern of TRPM4 was described [32]. TRPM4 protein was reported to be highly expressed in tumor buds, and high expression of TRPM4 was correlated with a higher number of tumor buds and an increased percentage of the infiltrative tumor border configuration. These small clusters of up to five cancer cells, called tumor buds, and an infiltrative tumor border configuration were correlated with lymphatic vessel invasion and lymph node metastasis in CRC [113,114,115], thereby linking TRPM4 expression to worse patient outcomes. The discrepancy between these three studies could be due to the type of samples or different sample sizes that were analyzed. One study analyzed cell lines, whereas the two other analyzed tissue samples. In addition, mRNA expression analysis of a homogenized sample can show a different image of the data, while tissue slices stained for protein expression provide a more heterogeneous display of the expression patterns in different subsections of the tissue.

In a study comparing the mRNA and protein expression levels of different TRPM family members in urinary bladder cancer (40 cancer tissue + 7 control samples), no differences in TRPM4 expression, compared to healthy controls, were detected [105]. In the same study, TRPM4 protein was only detected in the epithelial cells of the bladder in both the cancer and control groups.

A gene expression study of cervical cancer cases reported TRPM4 to be overexpressed in cervical cancer specimens compared to normal cervical epithelium [38]. TRPM4 expression was also analyzed in endometrial cancer with a public data-based expression analysis. Here, TRPM4 and two other genes (LMNB1 and KIAA1644) were identified as protective prognostic genes. In combination with six other genes (PHLDA2, GGH, ESPL1, FAM184A, LMNB1, and KIAA1644), TRPM4 performed well as a diagnostic prediction signature for endometrial cancer [106]. Moreover, a recent expression study of 491 endometrial cancer patients revealed that decreased TRPM4 mRNA expression is significantly correlated with a poor prognosis and reduced overall survival [116].

Lastly, TRPM4 mRNA was previously described in the human liver cancer cell lines, HepG2 and Huh-7 [117]. However, to date, the putative role of TRPM4 in liver cancer pathophysiology has not been investigated.

## 4. Functional Role of TRPM4 in Different Types of Cancer

### 4.1. Prostate Cancer

Prostate cancer is, thus far, the most studied type of cancer with regard to TRPM4 expression and function. In 2014, TRPM4 was described to be a cancer driver gene in androgen-independent PCa [118]. One year later, Holzmann et al. reported TRPM4 to be responsible for Ca^2+^-activated nonselective (CAN) current in PCa, as the large Na^+^ currents developed upon Ca^2+^ activation were decreased in PC3, LNCaP, and DU145 PCa cells, as well as primary human prostate epithelial cells (hPEC) with siRNA-based TRPM4 knockdown [41]. Moreover, fluorescence-based Fura-2 Ca^2+^ imaging experiments from two independent groups revealed changes in the Ca^2+^ entry pathways via SOCE in TRPM4 knockdown cells. While one study reported elevated levels of Ca^2+^ entry in TRPM4 siRNA-transfected DU145 and hPEC cells with no changes in PC3 cells [41], another study demonstrated decreased intracellular Ca^2+^ entry in PC3 TRPM4 knockdown cells [35]. The inconsistency between these two studies could be due to their different knockdown techniques. Whereas the first study was based on transient knockdown experiments, the second study used stable knockout cells, possible yielding compensatory effects due to the knockout. Nevertheless, the fact that TRPM4 is involved in the regulation of intracellular Ca^2+^ signaling in cancer cells is highly interesting, as Ca^2+^ oscillations and maintaining a cytosolic Ca^2+^ gradient in migratory cells were shown to contribute to cancer cell migration [119]. Indeed, several independent studies have shown that TRPM4 contributes to the migration and invasion of the PCa cell line PC3 [34,37,41]. Furthermore, Sagredo et al. showed that TRPM4 expression in PC3 cells can alter EMT [34], an important process for cancer cell migration and invasion. The downregulation of TRPM4 caused a shift in E-cadherin and N-cadherin expression levels and reduced the expression of Snail1, a well-known transcription factor of EMT markers. Migration was additionally shown to be decreased in DU145 TRPM4 knockout cells [110]. However, the potential involvement of Ca^2+^ in this mechanism still needs to be investigated. TRPM4 was also reported to be involved in the proliferation of DU145 [110] and PC3 [35] cells, whereby the latter was shown to be regulated by the activity of GSK-3β and β-catenin [35]. The downregulation of TRPM4 led to decreased nuclear β-catenin levels, leading to a decrease in its transcriptional activity. This decrease in TRPM4 expression further decreased the total β-catenin protein levels and activated GSK-3β, a negative regulator of Snail1, through phosphorylation [120]. Moreover, TRPM4 was recently described to be negatively regulated by microRNA-150 (miR150). The downregulation of TRPM4 or upregulation of miR150 resulted in decreased proliferation, the inactivation of β-catenin, a shift in the cell cycle, and the suppression of EMT of PC3 cells [37]. More recently, a role in cell adhesion was described for DU145 cells, where TRPM4 knockout led to decreased cell adhesion and rounder cells [110].

### 4.2. Colorectal Cancer

A recent study described the role of TRPM4 in the cancer hallmark functions of colorectal cancer. TRPM4 was shown to conduct large Na^+^ currents in the HCT116 colorectal cell line and to act as the main source of CAN current in these cells [32]. The same study also showed a tendency for decreased viability and proliferation, as well a shift in the cell cycle, of stable TRPM4 knockout cells. In addition, TRPM4 was suggested to contribute to decreased migration and invasion. Finally, TRPM4 ion conductivity was demonstrated to be essential for viability and cell cycle shift, suggesting that TRPM4 is a potential anticancer drug target for CRC.

### 4.3. Cervical Cancer

shRNA-mediated TRPM4 downregulation was shown to lead to decreased cell proliferation in the cervical-cancer-derived cell line HeLa [36]. In addition to decreased proliferation, changes in cell cycle distribution were observed. Cells with reduced TRPM4 expression displayed, like with CRC [32] and PCa [37] cells, a higher number of cells in the G1 phase along with a decreased percentage of cells in the S phase compared to the control transfected cells. In addition, cyclin D1 and survivin expression levels were decreased upon TRPM4 knockdown. Cyclin D1 is known to promote the transition from the G1 phase to the S phase and could potentially explain the observed decrease in proliferation after TRPM4 knockdown. Moreover, in line with the PCa study, TRPM4 knockdown in HeLa cells led to a reduction in total β-catenin expression via GSK-3β-dependent degradation. An immunofluorescence analysis also indicated the predominantly cytoplasmic expression of β-catenin and only low nuclear expression [121].

### 4.4. Endometrial Cancer

The role of TRPM4 in the cancer hallmark functions of endometrial cancer was recently studied. Contrary to the results described for many other cancer types, silencing of TRPM4 increased the cell viability and migration rate of AN3CA endometrial cancer cell line. The depletion of cells from TRPM4 leads to increased EMT progression, determined according to changes in EMT marker expressions (increased N-cadherin and vimentin expression along with decreased E-cadherin and cytokeratin expression in siTRPM4). Moreover, TRPM4 expression was found to be associated with the p53 and PI3K/AKT/mTOR signaling pathways, as the downregulation of TRPM4 leads to increased phosphorylation of PI3K, AKT, and mTOR, as well as decreased p53 expression levels [116].

### 4.5. Breast Cancer

The migration effect observed from KCTD5 is mediated through TRPM4 [39]. KCTD5 is a putative adaptor for Cullin3-E3 ubiquitin ligase and could contribute to TRPM4 turnover via ubiquitinoylation. In addition, KCTD5 regulates migration by altering Ca^2+^ signaling, possibly via TRPM4 or other ion channels and Rac1 activity [122,123]. Moreover, in a gene set enrichment analysis of three breast cancer gene expression profiling data sets, TRPM4 expression was shown to be associated with EMT gene sets and estrogen response gene sets [40].

### 4.6. Other Cancers

In acute myeloid leukemia (AML) patients and AML cell lines, expression of TRPM4 is significantly increased. In addition, the knockdown of TRPM4 inhibited proliferation and cell cycle progression through the AKT/GLI1/Cyclin D1 pathways [124]. For liver cancer, urinary bladder cancer, and DLBCL, to date, only expression studies have been performed. However, the role of TRPM4 in these cancers should be investigated at the cellular and molecular levels.

## 5. Potential as a Drug Target

Following overexpression in many tumor types and the multiple links to cancer hallmark functions, TRPM4 has been suggested to be a new potential anticancer drug target. Not only its expression pattern and links to different types of cancer but also the fact that TRPM4 is mostly expressed on the cell surface make TRPM4 an interesting target. Membrane proteins, such as ion channels and receptors, can be targeted by larger molecules, such as antibody-based drugs. However, antibodies are mostly unable to pass through the cell membrane. Small molecular inhibitors, on the other hand, can diffuse through the plasma membrane and thus interact with the intracellular binding sites of channels and receptors. Therefore, membrane proteins can be targeted by both antibodies and inhibitors. For studies in primary cells and animal models, potent and selective inhibitors are needed, but the lack of selective and potent inhibitors has made it difficult to evaluate TRPM4 as an anticancer drug target. The two most commonly used inhibitors for TRPM4, flufenamic acid and 9-phenanthrol, both show low potency and a lack of selectivity. Flufenamic acid was originally used as an anti-inflammatory drug before being considered as a cation channel inhibitor. However, flufenamic acid was later shown to have many off-targets among other ion channels [125], and 9-phenanthrol was shown to inhibit the Ca^2+^-activated Cl^-^ channel TMEM16A [126] and also activate the K_Ca_ 3.1 channels [127]. Moreover, it has also been reported that a third commonly used TRPM4 inhibitor, Glibenclamide, has an inhibitory effect on K^+^ channels [128,129].

Additionally, TRPM4′s broad expression is an important factor to consider. Since TRPM4 is widely expressed, it would be necessary to target any related drug therapy to a specific organ or type of cells to avoid unwanted systematic side effects. One possibility for such targeting would be to use caged blockers that are “opened and released” by organ-specific enzymes; one could also target TRPM4 with bispecific antibodies. The dual-targeting of TRPM4 in combination with any of its interaction partner proteins or tumor-specific membrane proteins with bispecific-antibodies carrying toxins could diminish the risk of unwanted side effects in other tissues of the body.

Recently, a TRPM4-specific antibody named M4P was described to inhibit TRPM4′s current by binding close to the channel pore [130]. M4P was shown to downregulate TRPM4 surface expression in rat models of stroke, and to inhibit hypoxia-induced cell swelling and reduce reperfusion injury in stroke recanalization. Moreover, M4P has anti-oncotic effects in neurons, astrocytes, and vascular endothelial cells under hypoxic conditions [131]. In addition, three new small molecular inhibitors of TRPM4—CBA ((4-chloro-2-(2-chlorophenoxy) acetamido)benzoic acid, also called compound 5), NBA (2-(1-naphthyloxyacetamido)-4-chloro-benzoic acid), and LBA (4-chloro-2-(2-(4-chloro-2-methylphenoxy)propanamide) benzoic acid))—were recently described [132,133]. CBA showed a solid inhibitory effect, both on HEK293 cells overexpressing TRPM4 and on PCa LNCaP cells endogenously expressing TRPM4, when analyzed with electrophysiological methods. CBA presented 10-fold stronger inhibition compared to 9-phenanthrol [133] and demonstrated selectivity over other TRP family members (TRPV1, TRPV3, TRPV6, TRPM5, TRPM7, and TRPM8). The effect of CBA and its two derivatives, NBA and LBA, was evaluated on endogenous TRPM4 in PCa LNCaP cells. CBA presented a low IC_50_ value of 1.1 ± 0.3 μM [133], an LBA of 0.74 ± 2.0 µM [110], and an NBA IC_50_ of 0.16 ± 2.4 µM [110] in LNCaP cells. In addition, these blockers were evaluated for their effect on proliferation and migration in the androgen-independent PCa cell line, DU145. Nevertheless, due to the incomplete blockage of TRPM4 currents (65% to 88% at 50 µM) in these cells, the effects on cellular functions were limited [110]. Recently, these TRPM4 inhibitors were optimized and investigated for their structural activity relationship (SAR). As a consequence, at least two new inhibitors, compound 8 and compound 9, were identified and will be investigated further [132]. Future studies will reveal if any of these TRPM4 blockers or the M4P antibody impact cancer hallmark functions of other types of cancer.

## 6. Conclusions and Perspectives

TRPM4 is a potential diagnostic marker for cancer progression, as a correlation between TRPM4 expression and worse diagnostic outcomes has been reported for breast cancer [39], DLBCL [33], and PCa [34,103]. In addition, TRPM4 was suggested as a protective diagnostic marker for endometrial cancer [106]. Moreover, TRPM4 has been linked to proliferation, migration, invasion, and EMT progression. However, despite numerous interesting studies on TRPM4′s expression and involvement in cancer, we are only beginning to understand TRPM4′s role in the pathophysiology of cancer. No studies on TRPM4 in cancer mouse models have been published. Further studies using xenograft mouse models and TRPM4-KO cells or specific TRPM4 inhibitor treatments could give a better overview of the role of TRPM4 in tumor development, tumor growth, and metastasis spread. In addition, these experiments could potentially give a better overview of the role of TRPM4 in the tumor microenvironment and its interactions with immune responses, cytokines, growth factors, etc.

Aside from the potential use of TRPM4 as an anticancer drug target, as discussed above, the question of whether ion conductivity is needed for cancer development and progression remains partially unanswered. Would it be enough to block TRPM4′s current to alter cancer progression, or would it be preferable to use TRPM4 as a tumor-specific target for the delivery of toxins? In the colorectal cancer cell line HCT116, ion conductivity was shown to be important for cell cycle and viability regulation [32]. However, further studies must be performed to determine if this is the case for all cancer types and whether the same mechanisms of TRPM4 action regulate other cancer hallmark functions.

Taken together, these studies shed light on the potential of TRPM4 as an anticancer target and tool for diagnostic purposes. Nevertheless, more studies are needed to expand our knowledge of the mechanisms behind TRPM4′s involvement in cancer development and progression.
